# Perioperative nursing care strategies in esophageal cancer surgery: a mini-review

**DOI:** 10.3389/fonc.2025.1633887

**Published:** 2025-09-23

**Authors:** Yu-feng Li, Ji-hong Tao, Chen-chen Wang, De-chun Su

**Affiliations:** ^1^ Department of Thoracic Surgery, Hongqi Hospital Affiliated to Mudanjiang Medical University, Mudanjiang, China; ^2^ General Surgery Department II, Hongqi Hospital Affiliated to Mudanjiang Medical University, Mudanjiang, China; ^3^ Department of Operating Room, Hongqi Hospital Affiliated to Mudanjiang Medical University, Mudanjiang, China

**Keywords:** esophageal cancer, surgery, perioperative care, nursing care, nursing practice

## Abstract

This study reviews contemporary clinical trials on perioperative nursing care management (PNCM) in esophageal cancer (EC) surgery. It evaluates four key phases of care: preoperative preparation (patient education and physiological optimization), intraoperative management (physiological monitoring and multidisciplinary team coordination), postoperative care (complication prevention and recovery), and follow-up strategies. Evidence indicates that structured PNCM protocols are associated with improved surgical outcomes. Key interventions include standardized preoperative education programs, intraoperative physiological monitoring protocols, postoperative multimodal analgesia, personalized nutritional supports, and structured rehabilitation pathways. These interventions have demonstrated clinical efficacy, reflected in reduced postoperative complications (especially pulmonary and anastomotic), improved quality-of-life measures, and increased 1-year survival rates. Integration with multidisciplinary surgical teams plays a critical role in optimizing patient outcomes. Future research should prioritize: (1) validating integrated care pathways, (2) personalizing interventions through precise risk stratification, and (3) incorporating technological advances in perioperative care. This review highlights the essential role of specialized perioperative nursing in determining EC surgery outcomes and offers an updated, evidence-based framework to guide both clinical practice and future research.

## 1 Introduction

Esophageal cancer (EC) remains a significant global health challenge, ranking as the seventh leading cause of cancer mortality worldwide (1, 2). It predominantly affects males over 55 years, with major risk factors including tobacco use, alcohol consumption, chronic gastroesophageal reflux, and dietary factors (3). Late diagnosis contributes to its poor prognosis, as most cases present at advanced stages. Despite surgical advances, EC management remains complex due to high perioperative complication rates (4).

Surgical resection represents the primary curative option for localized EC (5, 6). The procedure’s success depends on both surgical precision and comprehensive perioperative care, given the esophagus’s anatomical complexity. Nursing care plays a pivotal role throughout the surgical continuum (7, 8). From preoperative preparation to postoperative recovery, specialized nursing interventions address EC-specific challenges including nutritional support, pain management, and respiratory care (9, 10). Perioperative nursing care management (PNCM) has emerged as a critical component for improving outcomes, though current evidence requires systematic consolidation. Evidence demonstrates that optimized PNCM reduces complications through vigilant monitoring and early intervention (7–10).

Recent literature has identified several emerging trends in PNCM for EC that warrant greater emphasis in this review. Key advances over the past five years include the integration of Enhanced Recovery After Surgery (ERAS) pathways specifically tailored for EC, the application of telehealth and remote monitoring technologies to support postoperative recovery, and the implementation of multidisciplinary care models that have shown improvements in both survival and quality of life (8, 11, 12). Furthermore, novel predictive tools—such as risk stratification models incorporating perioperative risk factors—have been developed to guide individualized nursing interventions (13). Incorporating these developments enhances the contemporary relevance of PNCM strategies and identifies priority areas for future innovation. However, much of the existing research continues to reiterate established practices without adequately incorporating these recent advancements, thereby limiting its novelty. A more comprehensive synthesis that integrates cutting-edge approaches—such as ERAS protocols specifically adapted for esophagectomy, telehealth-enabled postoperative monitoring, and artificial intelligence–assisted risk prediction—would enhance the relevance of the present review and align it more closely with contemporary global trends in surgical oncology.

This review aims to advance the understanding of PNCM in EC surgery by explicitly addressing gaps identified in earlier literature, which has often placed limited emphasis on recent technological innovations and multidisciplinary integration. By integrating contemporary clinical evidence with emerging care models, the present study consolidates best practices and highlights evidence-based innovations—such as ERAS protocols tailored for esophagectomy, telehealth-assisted postoperative monitoring, and artificial intelligence–driven risk prediction systems. These approaches provide forward-looking strategies that can inform both clinical decision-making and health policy development. Collectively, these contributions distinguish this review from prior work and enhance its relevance and applicability in the context of modern surgical oncology.

## 2 Search strategy and study selection

### 2.1 Search strategy

A systematic literature search was conducted in PubMed (an international biomedical database) and the China National Knowledge Infrastructure (a leading Chinese academic database) from January 2020 to July 2025. The search employed the following keywords: “esophageal cancer”, “nursing care”, and “surgery”. Boolean operators (AND/OR) were applied to refine results, and search syntax was adapted for each database to ensure comprehensiveness.

### 2.2 Eligibility criteria

The eligibility criteria for this study were defined as follows. Inclusion criteria comprised: (1) randomized controlled trials, retrospective cohort studies, and observational studies examining PNCM in EC surgery patients; (2) studies involving adult patients (≥18 years) with histologically confirmed EC undergoing surgical resection; and (3) studies providing comprehensive descriptions of perioperative nursing interventions. The exclusion criteria were applied as follows: (1) duplicate publications or studies with overlapping patient cohorts to ensure data independence; (2) studies not exclusively focused on EC; (3) studies lacking specific perioperative nursing interventions; and (4) non-clinical trial publications, including reviews, case reports, conference abstracts, editorials, and commentaries.

### 2.3 Study selection

**Figure 1:**
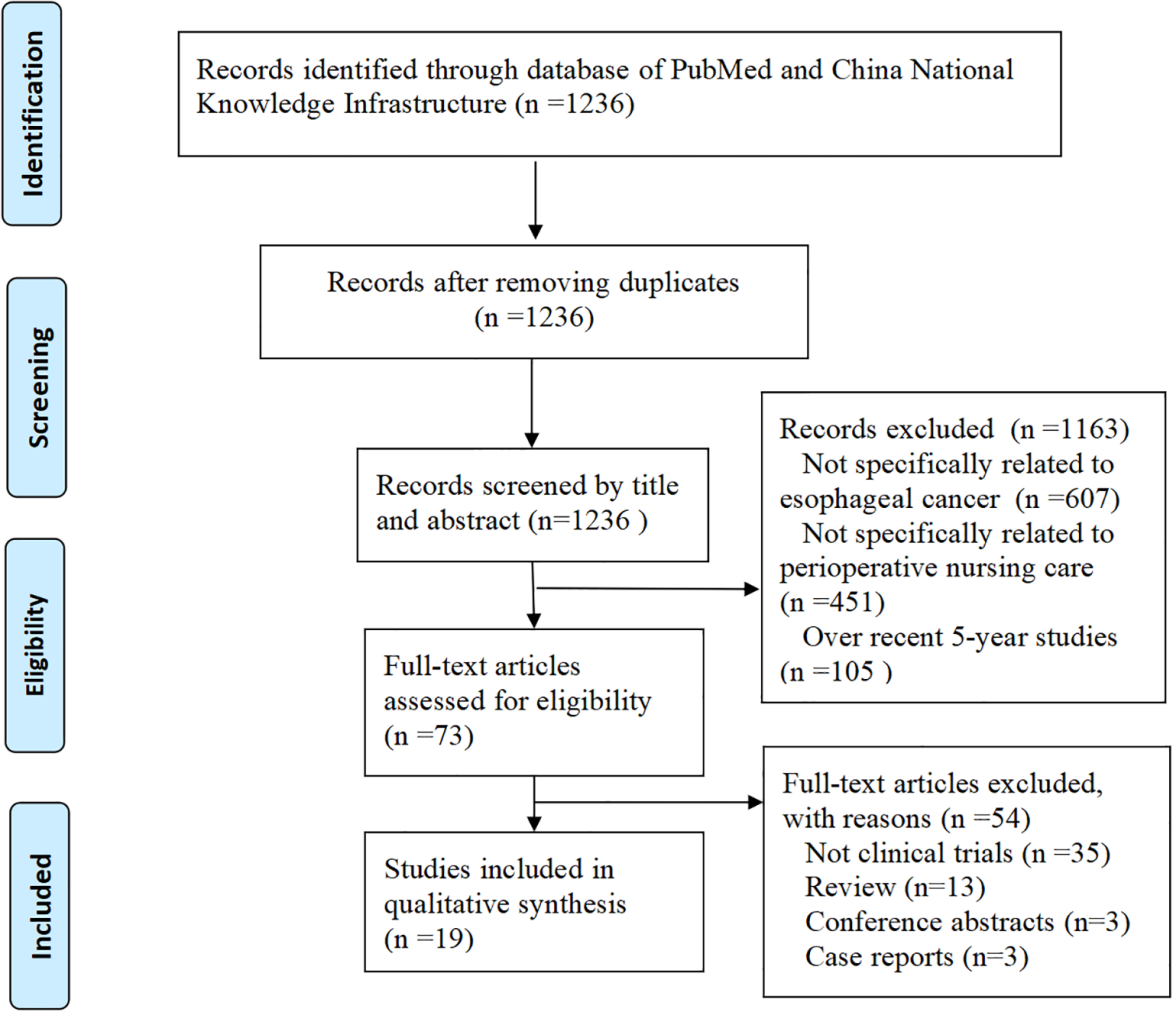
Flowchart of study selection.

The study selection process is summarized in **Figure 1**. A total of 1,236 articles were initially identified. After screening titles and abstracts, 1,163 records were excluded due to lack of relevance to EC (n = 607), perioperative nursing care (n = 451), or over recent 5-year publications (n = 105). The full texts of the remaining 73 studies were reviewed in detail. Of these, 54 studies were excluded for not involving clinical trials. Ultimately, 19 clinical trials met the inclusion criteria and were included in this study.

## 3 Advances in PNCM

### 3.1 Definition and framework of structured PNCM protocols

Structured PNCM protocols refer to standardized, evidence-based frameworks that guide nursing interventions throughout four critical phases: preoperative, intraoperative, postoperative, and follow-up (9). These protocols are designed through multidisciplinary collaboration to improve patient safety, ensure consistency in clinical practice, and optimize surgical outcomes, particularly in EC surgery (9). They serve as a cornerstone for quality improvement by streamlining workflows and providing measurable benchmarks for nursing care evaluation.

Unlike conventional approaches that rely on individualized clinical judgment, structured PNCM protocols promote uniformity in nursing interventions, thereby reducing variability and minimizing preventable complications (9). The following sections elaborate on the specific components and recent advances in each phase of PNCM.

### 3.2 Phase-specific implementation and advances in PNCM

Preoperative nursing: Recent studies show that structured preoperative nursing interventions significantly improve surgical readiness and perioperative outcomes in patients with EC (**Table 1**). Tailored educational programs, combined with multimedia resources, improve patients’ understanding of surgical procedures and postoperative expectations, thereby enhancing compliance and reducing anxiety (10, 14, 15). Psychological interventions, including mindfulness-based stress reduction and motivational nursing, alleviate preoperative anxiety and depression, fostering a positive mental state before surgery (16, 17). Preoperative implementation of ERAS protocols, including optimized nutritional preparation and early mobilization planning, is associated with improved postoperative recovery and reduced complication rates (18, 19).

**Table 1:** Summary of clinical studies on perioperative nursing care managements for patients with EC surgery.

Study	Patients	Study type	Sample size	Modality	Main findings
Liang YH, 2025 (10)	ECUS	Observation study	98	Nursing care model	Enhanced perioperative nursing for EC surgery reduces complications, shortens hospital stays, and lowers costs. Standardized protocols can improve outcomes and patient satisfaction.
Pan L. 2020 (14)	ECUS	Observation study	76	Clinical nursing care	Clinical nursing care, including respiratory care and dietary guidance, are crucial for the success of EC surgery.
Zhang YQ. 2020 (15)	ECUS	Observation study	102	Comprehensive nursing care	Comprehensive nursing care significantly improves emotional states and accelerates recovery.
Han WH, 2024 (16)	ECUS	RCT	100	Motivational + ERAS nursing care	Combined motivational and ERAS nursing improved nutritional indicators, reduced pain and anxiety/depression scores, and lowered complication rate vs. control.
Wang Q, 2024 (17)	ECUS	RCT	80	Mindfulness-based stress reduction nursing care	Reduced perioperative anxiety and depression, increased nursing satisfaction compared with conventional care.
Zhang YJ, 2025 (18)	ECUS	RCT	120	ERAS nursing care	ERAS significantly shortened time to first flatus, defecation, oral intake, reduced hospital stay and complications; improved self-care ability and QOL scores compared with conventional nursing.
Gao L, 2025 (19)	Elderly ECUS	RCT	76	ERAS nursing care	ERAS accelerated recovery, reduced pain, decreased complications, and alleviated negative emotions in elderly EC patients compared with conventional care.
Jiang RR, 2024 (20)	ECUS (robot-assisted)	Observation study	150	ERAS concept nursing care	Reduced complications and improved QOL and nutritional status in PNCM of robot-assisted EC surgery.
Shen GH, 2020 (21)	ECUS	RCT	70	ERAS nursing care	Reduced surgery, extubation, and hospital times; lower drainage volume; higher satisfaction vs. control.
Li WY, 2020 (22)	ECUS	RCT	70	Clinical nursing pathway	Higher satisfaction, shorter hospital and bed rest times, lower complication rate vs. control.
Chen LX, 2022 (23)	ECUS	RCT	100	Comfort nursing care	Shortened drainage, bed rest, and hospital stay; reduced anxiety, depression, pain, and improved sleep and QOL.
Feng Y, 2023 (24)	ECUS	RCT	80	Refined nutritional support nursing care	Improved nutritional indicators, QOL, and nursing satisfaction; reduced adverse reactions and negative emotions.
Han L, 2022 (25)	ECUS	Retrospective study	101	Refined nutritional support nursing care	Improved nutritional indicators; reduced complications; enhanced QOL scores.
Zhao SZ, 2022 (26)	ECUS	RCT	80	ERAS concept nursing care	Improved treatment compliance and QOL; reduced postoperative complications.
Luo ZH, 2021 (27)	ECUS	RCT	130	ERAS nursing care	Reduced anxiety/depression, promoted gastrointestinal function recovery, shortened hospital stay, and improved satisfaction.
Zhu SJ, 2020 (28)	ECUS	RCT	74	ERAS nursing care	Reduced complications and anxiety scores; shortened hospital stay.
Wang HY, 2022 (29)	ECUS	RCT	114	Evidence-based nursing care	Reduced pain and stress responses; improved prognosis; decreased complication rate vs. conventional nursing.
Yang CM, 2024 (30)	ECUS	RCT	113	Clinical nursing pathway	Lower complication rate, improved clinical indicators, QOL scores, and nursing satisfaction vs. control.
Ma PN, 2020 (31)	ECUS	Observation study	60	ERAS nursing care	Shortened recovery times, reduced pain, lowered complication rate compared with conventional care.

EC, esophageal cancer; ECUS, EC undergoing surgery; ERAS, Enhanced Recovery After Surgery; RCT, randomized controlled trial; QOL, quality of life.

Intraoperative nursing: Intraoperative nursing focuses on optimizing patient safety, procedural efficiency, and interdisciplinary communication (**Table 1**). ERAS-based approaches emphasize continuous monitoring of vital signs, meticulous anesthesia management, and strict aseptic techniques to reduce infection risk. Standardized intraoperative briefings and debriefings enhance coordination among surgical team members, allowing rapid adaptation to patient status changes (20). Integrating clinical nursing pathways improves intraoperative workflow and reduces surgery and extubation times (21, 22).

Postoperative nursing: Postoperative nursing has advanced significantly in pain management, nutritional support, and complication prevention (**Table 1**). Multimodal analgesia strategies that minimize opioid use reduce pain intensity and side effects (16, 23). Refined nutritional support pathways improve serum albumin, prealbumin, and muscle circumference, promoting wound healing and functional recovery (24, 25). Comfort nursing and ERAS protocols shorten drainage duration and hospital stay, lower complication rates, and improve quality of life (26–28). Evidence-based nursing interventions mitigate stress responses, reduce gastrointestinal symptoms, and improve overall prognosis (29).

Rehabilitation and follow-up: Rehabilitation nursing emphasizes early mobilization, progressive physical activity, and comprehensive follow-up strategies (**Table 1**). Clinical nursing pathways and ERAS protocols promote earlier ambulation, shorten hospitalization, and improve patient satisfaction (18, 30, 31). Long-term follow-up integrates physical rehabilitation with psychological counseling and social support, ensuring sustained improvements in functional independence and quality of life (10, 20).

In summary, these phase-specific advances in PNCM—supported by high-quality randomized controlled trials and observational studies—underscore the critical role of evidence-based nursing in improving perioperative outcomes for EC patients (**Table 1**). Integrating ERAS protocols, personalized education, psychological interventions, and refined nutritional care across all perioperative phases reduces complications, shortens recovery times, and enhances patient satisfaction.

## 4 Impact of improved PNCM on patient outcomes

### 4.1 Surgical outcomes

Enhanced PNCM has markedly improved surgical success rates and reduced complication rates in patients undergoing EC surgery (9, 10). Thorough preoperative assessments ensure optimal surgical readiness, minimizing risks such as bleeding and hemodynamic instability (9, 10). Intraoperatively, nurses’ precise monitoring and regulation of vital physiological parameters play a key role in ensuring patient safety (10). Postoperative use of advanced wound care and infection control protocols significantly reduces complications, leading to faster recovery and better surgical outcomes (10, 20). Beyond clinical outcomes, these findings offer practical guidance, especially in resource-constrained settings (9). The study shows that optimized nursing interventions enhance both surgical and postoperative outcomes, providing actionable insights for clinical practice (10, 16). For example, Liang et al. reported that the implementation of an enhanced perioperative nursing model significantly reduced the postoperative complication rate to 6.12%, compared with 34.69% in the conventional care group (χ² = 9.800, *P* < 0.05) (10). Patients receiving enhanced care began ambulation an average of 1.38 days earlier and had their mean postoperative hospital stay shortened by approximately 3 days (t = −5.658, *P* < 0.05) (10).

### 4.2 Patient satisfaction

Enhanced nursing care, especially in education, communication, and psychological support, is closely linked to higher patient satisfaction and improved postoperative quality of life (10, 18–27, 30). Clear communication helps patients understand procedures and expected outcomes, thereby reducing anxiety and building trust (10, 16, 17, 19, 23, 24, 27, 28). Individualized care, such as tailored pain relief and nutritional planning, enhances comfort and overall satisfaction during recovery (10, 17, 23, 24). Research consistently shows that patients who feel well-supported report greater satisfaction and improved quality of life post-surgery. In Liang’s study, the nursing satisfaction rate in the enhanced care group reached 93.98%, significantly exceeding the 87.67% observed in the conventional care group (χ² = 4.210, *P* < 0.05), underscoring the measurable impact of structured nursing interventions on patient-perceived quality of care (10). Moreover, these findings on patient satisfaction provide important insights for nursing education (10, 17, 21, 22, 24, 27, 30). They can inform curriculum development and training programs to enhance nurses’ communication and psychological care skills.

### 4.3 Survival rates

Advanced nursing care is critical not only for postoperative recovery, but also for improving short- and long-term survival (10, 16–19, 21, 22, 24, 29–31). Efficient preoperative preparation and comprehensive PNCM help shorten hospital stays and reduce readmissions, both of which are key predictors of short-term survival (10, 18, 21–23, 27, 28). Consistent, long-term follow-up care is essential for improving long-term survival by enabling routine health monitoring, facilitating early detection of complications or cancer recurrence, and ensuring timely intervention (10, 16, 18–20, 22, 25, 26, 28–31). Furthermore, among patients undergoing robot-assisted esophagectomy, an ERAS–based perioperative nursing approach significantly reduced postoperative complication rates and improved quality of life as well as nutritional status compared with standard care (*P* < 0.05) (20), suggesting that integrated, protocol-driven nursing care can yield survival benefits through both complication prevention and functional recovery support.

In summary, the advancement of PNCM in EC surgery has clearly improved surgical outcomes, patient satisfaction, and survival rates. This highlights nursing’s essential role in comprehensive cancer care and underscores the importance of continuous professional development to maintain these improvements.

## 5 Challenges and limitations

### 5.1 Challenges in implementing advanced PNCM

Implementing advanced PNCM for EC patients faces multiple challenges that limit widespread adoption (32–34). Logistically, integrating new protocols and technologies into existing healthcare systems is complex and often requires major workflow adjustments and interdepartmental coordination. Such integration typically involves extensive training and may temporarily disrupt routine care, reducing efficiency in the early phases (32, 33). From an educational perspective, keeping nurses proficient in updated practices requires ongoing professional development. However, regional disparities in training opportunities and educational resources may result in uneven care quality. In low-resource settings, shortages of skilled staff, medical technology, and funding severely hinder the implementation of advanced nursing care, compromising treatment efficacy (32–34).

### 5.2 Limitations of current studies

Research on PNCM for EC surgery has several limitations that must be addressed to improve the validity and applicability of results. A common issue is the small sample sizes, which limit the generalizability of findings to larger populations (31). The lack of longitudinal studies hampers the evaluation of long-term effects, as most research focuses only on immediate postoperative outcomes (17, 19, 22, 28, 31). Methodological inconsistency, including reliance on observational or non-randomized designs, introduces bias and may distort outcomes (10, 14, 15, 31). The underrepresentation of low-resource settings limits understanding of how advanced nursing practices perform under such conditions. The lack of standardized reporting across studies impedes systematic comparison and synthesis, which is critical for formulating evidence-based guidelines (17, 21, 22, 28, 31).

In summary, despite notable progress in PNCM for EC surgery, implementation remains hindered by logistical, educational, and resource-based barriers. Moreover, methodological limitations and underrepresentation in current research must be addressed to strengthen the reliability and generalizability of evidence. Overcoming these challenges is essential to advance PNCM and improve patient outcomes in EC care.

## 6 Future directions

### 6.1 Potential areas for future research

In addition to advancing clinical nursing practice, this study offers substantial educational value. By examining personalized and multidisciplinary care models, the study provides meaningful guidance for nursing curriculum development (35). Future research should explore how these models can be integrated into nursing education to enhance the clinical competencies of nursing professionals.

As nursing care evolves, several key areas warrant further investigation to improve outcomes for EC surgery patients. Studies on integrated care models that include medical, nutritional, and psychological support are essential, as they may significantly improve holistic patient outcomes (8, 11). The effectiveness of multidisciplinary teams, involving surgeons, nurses, and mental health professionals, requires rigorous evaluation to assess their impact on recovery and satisfaction (8, 11). Additionally, personalized nursing care tailored to individual factors—such as genetics, comorbidities, and social context—is a promising research area (36). Understanding how these factors affect surgical recovery could help develop more targeted and effective care strategies. Research on advanced pain management and optimized nutritional support in postoperative care may lead to improved recovery and shorter hospital stays.

### 6.2 Technological advancements

Technological innovations have the potential to significantly transform PNCM for patients undergoing EC surgery (37). Telehealth enables remote monitoring and consultation, supporting continuous care after discharge and potentially reducing readmission rates (37). The use of data analytics and artificial intelligence in PNCM warrants further study. They can predict outcomes, personalize care plans using predictive models, and identify high-risk patients for early intervention. Immersive technologies such as virtual and augmented reality offer promising applications in nursing education, providing realistic, controlled environments for procedural training that improve staff preparedness and patient safety.

In summary, future research should prioritize evaluating integrated and personalized care models, investigate emerging technologies to improve patient care, and assess the impact of innovative training methods on nursing practice quality. These efforts are essential for advancing nursing in surgical oncology and improving patient outcomes.

## 7 Summary

This study summarizes key advancements in PNCM for patients with EC undergoing surgery. Notable improvements in preoperative preparation, intraoperative monitoring, and postoperative care have collectively contributed to improved surgical outcomes and reduced complication rates. Innovations such as comprehensive patient education, optimized pain management, and individualized nutritional support have been associated with higher patient satisfaction and improved postoperative quality of life.

In addition, emerging approaches, including telehealth-assisted postoperative monitoring, ERAS protocols tailored for esophagectomy, and artificial intelligence-based risk prediction tools, represent promising directions for further optimizing PNCM. To maximize these benefits, evidence-based practices should be integrated into nursing education and continuous professional development programs. Such integration will better prepare equip nurses to address the complex needs of surgical patients and improve clinical outcomes. Incorporating advanced nursing strategies into routine clinical practice is expected to yield substantial improvements in patient outcomes. Nursing teams should adopt evidence-based approaches that emphasize patient education, psychosocial support, and interdisciplinary coordination. Ongoing professional development is essential to maintaining nursing proficiency in both established therapeutic techniques and emerging technologies in surgical oncology. Continuous education enables nurses to adapt effectively to the evolving challenges of surgical patient care.
